# Supplementary material for: Bovine mastitis epidemiology: Prevalence, risk factors, control program gaps and biosecurity recommendations to improve animal health in the Rwandan smallholder dairy farms
Source: PLoS One. 2026 Mar 6;21(3):e0329250. doi: 10.1371/journal.pone.0329250 (PMC12965528; doi:10.1371/journal.pone.0329250)
Supplement: S2 Appendix — (DOCX) [file pone.0329250.s002.docx]

| Format sheet used during mastitis screening data collection. | | | | | |
| --- | --- | --- | --- | --- | --- |
| **Description** | **Parameters** | | | | |
| Cow ID or its name (if applicable) |  | | | | |
| District | **1.** Nyabihu **2.** Musanze | | | | |
| Grazing system | **1.** Zero grazing **2.** Open grazing | | | | |
| Cow Age | **1.** Young cow (1 to 6 years), **2.** Old cow (7 and over) | | | | |
| Breed | **1.** Exotic cow (pure breed) **2.** Cross breed **3.** Ankole | | | |  |
| Parity | **1.** Early (1 and 2 times) 2**.** Middle (2 to 5 times) **3.** Later (6 times and over) | | | |  |
| Stage of lactation | **1.** Early stage (1 to 2 months) **2.** Middle stage (2 to 6 months) **3.** Advanced stage (7 months and over) | | | |  |
| Presence of teats ulcerations and cracks | 1. Yes **2.** No | | | |  |
| Cow Hygiene | **1.** Very dirty (the entire udder, back legs, tail, and belly are muddy filled). **2.** Fairly or moderately dirty (a part of udder and small scale of back, tail and belly muddy). **3.** Slightly dirty (a very small traces of mud fill the udder other part remaining clean)  **4.** Not dirty (any trace of mud exists on the udder and the rest parts). | | | |  |
| **Sub-clinical mastitis** | | | | | |
| CMT Results at **quarter level** | Quarter 1  (Front Left) | Quarter 2  (Front Right) | Quarter 3  (Back Left) | Quarter 4  (Back right) | |
|  |  |  |  |  | |
| **Clinical mastitis** | | | | | |
| Check clinical mastitis | 1. Enlarged udder (Swelling) 2. Redness 3. Change of udder and teats symmetry 4. Pain of the udder/teats during palpation 5. Watery milk 6. Milk with pus 7. Milk with clots, 8. Blind quarters (amabere yazibye) | | | |  |
