# Supplementary material for: Bovine mastitis epidemiology: Prevalence, risk factors, control program gaps and biosecurity recommendations to improve animal health in the Rwandan smallholder dairy farms
Source: PLoS One. 2026 Mar 6;21(3):e0329250. doi: 10.1371/journal.pone.0329250 (PMC12965528; doi:10.1371/journal.pone.0329250)
Supplement: S3 Appendix — (DOCX) [file pone.0329250.s003.docx]

**QUESTIONNAIRE FOR INDIVIDUAL FARMERS**

**Consent**

Do you provide consent to participate in this Interview? Yes No

**SECTION A: BASIC INFORMATION Date of Interview: -----------------------**

**A1. Demographic information**

| A1.1 Name (optional) |  |
| --- | --- |
| A1.2. Sex |  |
| A1.3. Telephone no |  |
| A1.4. Village: | Cell: |
| A1.5. Sector: | District: |
| A1.6. Age of the respondent (years) | **……………** |
| A1.7. Type of household | (**1**) Male-headed (husband & wife) (**2**)- Female- headed (husband & wife) (**3**) Male-headed (husband no wife), (**4**)- Female-headed (Wife no husband) (**5**)- Child-headed (No father and mother) -- |
| A1.8. Number of household members/persons in the following age categories by sex | - Below 18 years of age (Male……...; Female: …………...)  - 18 to 50 years of age (Male……...; Female: …………...)  - Above 50 years of age (Male……...; Female: …………...) |
| A1.9. Can you read and write? | 1. Yes............2. No ……… |
| A1.10. If yes to A9, what is your highest education level? (Enter years of school) | **1.** Primary--------------------**2.** Secondary--------------------  **3.** College/Higher Education---------**4.** No formal education------ |
| A1.11. Have you received any assistance from dairy development projects? 1. Yes 2. No | If yes, what project? What assistance did you receive? |
| A1.12. Are you a member of LFFS group: 1. Yes 2. No | If yes, name of LFFS: __________________________ |
| A1.13. Are you a member of a cooperative: 1. Yes 2. No | If yes, name of the cooperative: __________________________ |

**A.2. Dairy Production Information**

| **Questions** | **Currently** |
| --- | --- |
| A2.1. What is the total number of cows do you have?  Number: ……… | Females (in milking) ……Female in dry period…...Bulls……., Calves…. |
| A2.2. What breed of animals do you keep (**1**) Ankole…. (**2**) Crossbreed…. (**3**) Friesian…. (**4**) Jersey…. (**5**) Brown Swiss |  |
| A2.3. How many cows are in milk? |  |
| A2.4. How many total liters are they currently produce (ing) per day? |  |

**SECTION B: MANAGEMENT PRACTICES**

**B1. Milking and milk handling practices**

| **Questions** | **Currently** |
| --- | --- |
| B1.1. How do you milk your cows? 1. hand milk 2. Milking machine. 3. Both |  |
| B1.2. If hand milk, who milks your cattle? 1. Husband 2. Wife 3. Child 4. Worker 5. Other (specify) ……………………………………… |  |
| B1.3. If hand milk, how long does it take to milk a cow?  1. Less 7 mins 2. Between 8 to 15 mins 3. More than 15 min |  |
| B1.4. If hand milk, do you let calves suck the miking cow before, within and after milking? 1. Yes…. 2. No…… |  |
| B1.5. If hand milk, do you keep milk in the udder for calves to suck after milking?  1. Yes…...2. No…. |  |
| B1.6. If a milking machine is used, how long does it take to milk a cow?  1. Less 7 mins 2. Between 8 to 15 mins 3. More than 15 min |  |
| B1.7. Do you milk cows when you are suffering from contagious diseases like diarrhea or typhoid? 1. Yes 2. No 3. Don’t know |  |
| B1.8. Do you milk your cows while smoking, coughing, spitting, and sneezing? 1. Yes 2. No 3. Don’t know |  |
| B1.9. Do you sell your milk at an MCP/MCC 1. Yes 2. No |  |
| B1.10. If yes, how long distance (Km) does it take you to deliver the milk at the MCP/MCC after milking? |  |
| B1.10. How many times in the past 12 months have buyers rejected your milk due to poor quality? (1) Several times (Twice a month) ……... (2) Rarely (Once in 3 months) …………… 3) Never happened……………… |  |
| B1.11. What containers are used to handle milk or milking.  1. Plastic, 2. Aluminum 3. stainless steel containers |  |
| B1.12. If there is a time lag between milking time and disposal of milk?  1. Yes……. 2. No……...  B1.13. How do you preserve your milk?  (i)-Refrigerating/Chilling----------(ii)-Cold water ---------(iii)-Boiling ----------(iv)-Using preservative like herbs ------------- (v) Others (Specify) ----------------- |  |

**B2. Hygienic milk handling**

| **Questions** | **Currently** |
| --- | --- |
| B2.1. Is there any source of potable water on the farm? 1. Yes 2. No |  |
| B2.2. What is the principal source of water used on your farm?  1. Borehole 2. Dam 3. Surface water (river, lake...)  4. Tap water/National water supply (EWSA) 5. Rainwater 6. Others (specify)…... |  |
| B2.3. How do you normally prepare your cow’s udder before milking? 1. Clean the udder with luke-warm water 2. Apply milking jelly/salve 3. Apply both 4. Direct milking (none of above) (5)-Others (Specify) --------- |  |
| B2.4. Do you dry/clean the udder with a tower or cloth before milking?  1. Yes……. 2. No……... |  |
| B3.5. How often do you wash/clean your cloth or towel used in milking?  1. Clean twice a day 2. Once a day 3. Once a week 4. Over a week |  |
| B2.6. How does the milkers normally prepare themselves before milking? 1. Warm water 2. Cold water 3. Warm water and detergent 4. Cold water and detergent 5. Direct milking (none) |  |
| B2.7. If detergent is used, what type of detergent?  ………………… |  |
| B2.8. If a milking machine is used, do you wash your machine with detergent?  1. Yes ……… 2. No……… |  |
| B2.9. If yes what detergent used?.............................. |  |
| B2.10. If a milking machine is used, do you wash your machine with Teat Liners?  1. Yes … 2. No……… |  |
| B2.8. Who cleans milking utensils/equipment?  1. Husband 2. Wife 3. Child 4. Worker 5. Other (specify) …………… |  |
| B2.9. How do you clean milking utensils/equipment? 1. Clean with warm only 2. Clean with cold water only 3. Warm water and detergent  4. Cold water and detergent (5)-Others (Specify) --------------------- |  |

**B3. Mastitis control practices**

| **Questions** | **Currently** |
| --- | --- |
| B3.1. Do you know what mastitis is? 1. Yes…. 2. No…... |  |
| B3.2. If yes B.2.1, did you ever experience any cases of mastitis during the milking period at your farm? 1. Yes…. 2. No…... |  |
| B3.3. If yes B.2.2, How often does your herd experience mastitis? 1. Once a week 2. Once a month 3. Once 6 month 4. Once a year |  |
| B3.4. Do you check for mastitis before milking (Teat striping)? 1. Yes….2. No…...    If yes, how?  1) Check for pus  2) Check for milk discoloration & blood clots in foremilk  3) Check watery milk.  4) Others (Specify)………………….  5) Not done |  |
| B3.5. What equipment do you use to check/screen for mastitis before milking?   1. CMT - Somatic Cell Count (SCC) 2. Use of strip cup 3. A cup (igikombe gisanzwe cya plastic cg icyuma) 4. Others (e.g., UdderCheck, Draminski, etc) …………... 5. None |  |
| B3.6. If yes, how often do you screen for mastitis?  1. Everyday 2. Once a week 3. Once a month and over 4. Never done |  |
| B3.7. When do you milk mastitic cows?  1) Milk them first (before milking other cows)  2) Between milkings  3) Last |  |
| B3.8. Do you treat mastitic cows? 1. Yes….2. No…. |  |
| B3.9. If yes, what kind of drugs did/do you use to treat mastitis?  1) Intra-mammary antibiotics  2) Antibiotics by injection  3) Traditional methods  4) Other method………………………, 5) I don’t know |  |
| B3.10 Who usually treats the animal (administer the medicine) for mastitis?  1. Veterinarian 2. Myself/My cow-keeper 2. A neighboring farmer |  |
| B3.11. If systemic injection is used; for how many days was/is the cow treated/ injected with the medicine?  1. Single injection 2. Twice 3. Three times 4. Don’t know |  |
| B3.12. If intramammary infusion is used; for how many days was/is the cow treated/ injected with the medicine?  1. Single treatment 2. Twice 3. Three times 4. Don’t know |  |
| B3.13. Did/do you get a prescription from the veterinarian? 1. Yes….2. No…. |  |
| B3.14. Do you discontinue therapy once the symptoms subside?   1. Yes…………...No………... |  |
| B3.15. What do you do with milk from animals undergoing antibiotic treatment?  1) Sold  2) Drink/consume  3) Withheld/Discarded  4) Feed calves  5) Other (describe)……………… |  |
| B3.16. Do you do pre-teat dipping? 1. Yes………2. No…… |  |
| B3.17. If yes B3.16, what kind of teat-dip solutions do you use? 1. Iodine  2. Chlorine dioxide 3. Others (specify)……………. |  |
| B3.18. Do you do post-teat dipping? 1. Yes…..2. No…….. |  |
| B3.19. If yes B3.18, what kind of teat-dip solutions did/do you use? 1. Iodine  2. Chlorine dioxide 3. Others (specify)……………. |  |
| B3.20. Do you know what dry-cow therapy is? 1. Yes…...2. No…... |  |
| B3.21. If yes B3.20, do you practice dry cow therapy? 1. Yes…...2. No…. |  |
| B3.22. If yes B3.21, what drug/medicine(s) do often use for dry therapy?................................................ |  |

**B4. Farming and waste management practices**

| **Questions** | **Currently** |
| --- | --- |
| B4.1. Production system: 1. Zero grazing 2. Open grazing |  |
| B4.2. If it is zero grazing, do you have a cow shed?  1. Yes 2. No |  |
| B4.3. Does your household have a manure pit 1. Yes ____ 2. No ___ |  |
| B4.4. Does your household have compost 1. Yes ___ 2. No _____ |  |
| B4.5. Does your household have a water harvesting tank 1. Yes ___2. No __ |  |
| B4.6. If it is open grazing, are all species mixed?  1. Yes 2. No |  |
| B4.7. Within the year, has your cattle experienced injury including cuts/lacerations/punctures, bruises, bone fractures, muscle strains/sprains, etc.?  1. Yes…... 2. No……, Specify…………………………………... |  |
| B4.8. If yes on 4.7, how many times did this happen? Specify……………. |  |
| B4.9. Do you keep farm records? 1. Yes_____ 2. No_____ |  |
| B4.10. If yes, what kind of daily dairy farm records kept at the farm, for example, (check the book for any record)  a) Liters of milk produced  b) Animal health (diseases)  c) Sales records  c) AI records  d) Staff payment  e) Payment for agro vet services  f) Payments for inputs  g) Other………………………….. |  |

**B5. Environmental management**

| **Questions** | **Currently** |
| --- | --- |
| B5.1. If zero grazing, do calves live in the same compartment as milking cows? 1. Yes 2. No |  |
| B5.2. If zero grazing, is the milking area separate from other rearing areas? 1. Yes 2. No |  |
| B5.3. If zero grazing, is there adequate lighting in the milking shed? 1. Yes 2. No |  |
| B5.4. If zero grazing, does the milking shed have a slope & well drained? 1. Yes 2. No |  |
| B5.5. If zero grazing, how often do you change bedding or clean the lying area to remove organic matter? 1. Everyday 2. Less one week 3. Between 1 week- two weeks  4. Once a month or over |  |
| B5.6. If open grazing, do calves live in the same area as milking cows? 1. Yes 2. No |  |
| B5.7. If open grazing, is the milking area separate from the grazing areas? 1. Yes 2. No |  |
| B5.8. If open grazing, is there adequate lighting in the milk area? 1. Yes 2. No |  |
| B5.9. How long does it take a cow to lay down after milking?  1. Less than 30 mins 2. Between 30 mins-1 hour 3. More than an hour 4. Don’t know |  |

**B6. Access to services (vet and trainings)**

| **Questions** | **Currently** |
| --- | --- |
| B6.1. Are private veterinarians available at any time you seek them? 1. Yes 2. No |  |
| B6.2. Are public veterinarians (e.g., sector or district) available at any time you seek them? 1. Yes 2. No |  |
| B6.3. How often do you interact with vets or how often do vets visit your farm? 1. Everyday 2. At least once a week 4. At least once a month 5. Over a month 6. Never |  |
| B6.3. Do you have access to training? 1. Yes 2. No |  |
| B6.4. Who trained/trains you? Specify …………………………………. |  |
| B6.5. How often do you get training (times in a year)? …………… |  |
| B6.6. On what topics were/are you trained on? Specify……………………... |  |
| B6.7. Do you realize any change in mastitis after training? |  |
| B6.8. If yes, what? Specify………………………………………………………. |  |
| B6.9. What is the most important training topic did you find more useful?  Specify……………………………………………… |  |

**B7. Other general questions**

| **Questions** | **Currently** |
| --- | --- |
| B7.1. What were/are the major cattle diseases on your farm?  (1) ECF & other tick-borne diseases (TBDs) (2) Foot and Mouth Disease (FMD) (3) Abortion (4) Mastitis (5) Worms (6) Lameness (7) RVF 8. LSD 9. Others (Specify)……… |  |
| B7.2. What disease control measures did/do you practice & how often weekly/monthly/annually? |  |
| 1) Spraying against ticks |  |
| 2) Deworming |  |
| 3) Vaccination |  |
| 4) Mastitis control: |  |
| B7.3 What diseases vectors are likely frequent at your farm?   1. Ticks 2. Mosquitoes 3. Others (specify)………………………… |  |
| 7.4. How often do you spray to protect against vectors? 1. once a week 2. Twice a week 3. Over 2 weeks |  |
| B7.3. How much money do you spend on a) spraying against per week/month/year……………………… |  |
| B7.4. How much did/do you spend on deworming per week/month/year?............... |  |
| B7.5. How much did/do you spend vaccination per month/year? ………………. |  |
| B7.6. What type of feeds do you give your cows?  a) Forage  b) Legumes  c) Grains  d) Farm-made concentrates  e) Water to satisfy cow need  f) Purchased concentrates |  |
| B7.7. Do you use molasses to feed cows? 1. Yes….2. No…... |  |
