# Supplementary material for: Bovine mastitis epidemiology: Prevalence, risk factors, control program gaps and biosecurity recommendations to improve animal health in the Rwandan smallholder dairy farms
Source: PLoS One. 2026 Mar 6;21(3):e0329250. doi: 10.1371/journal.pone.0329250 (PMC12965528; doi:10.1371/journal.pone.0329250)
Supplement: Attachment — Submitted filename: General Assessment.pdf [file pone.0329250.s004.pdf]

General Assessment

The manuscript is well-written, comprehensive, and addresses a highly relevant topic in veterinary public health and dairy production. The study is methodologically sound, the sample size is appropriate, and the results are clearly presented with useful statistical analyses. The discussion contextualizes findings well within regional and global literature. The conclusions are logical, with practical recommendations for policy and practice.

I recommend the manuscript for publication **after minor revision**.

Strengths

1. **Relevance** – Focuses on mastitis in smallholder systems in Rwanda, where limited epidemiological data exist.
2. **Study design** – Adequate sample size (411 farms) with stratified random sampling strengthens representativeness.
3. **Statistical analysis** – Logistic regression is appropriate; results are supported by confidence intervals and p-values.
4. **Clarity** – Clear structure with good integration of intrinsic, extrinsic, and management-related risk factors.
5. **Practical value** – Recommendations on biosecurity and national control programs have direct policy implications.

Areas for Minor Improvement

1. **Clarity and conciseness**
    - Some sentences are long and could be made more concise (particularly in the *Introduction* and *Discussion*).
    - Example: Line 40–42 of the Abstract could be shortened for impact.
2. **Consistency in terminology**
    - Use uniform terms for *cow hygiene* (e.g., “very dirty,” “fairly dirty”) — currently mixed with “muddy filled,” which may confuse readers.
    - “Mastitis control variants” (Abstract) is unclear. Consider rephrasing to “mastitis control measures or interventions.”
3. **Statistical reporting**

- Some odds ratios have extremely wide confidence intervals (e.g., OR = 11.02; 95% CI: 1.449–490.310). Mention this limitation briefly in the *Discussion* to show cautious interpretation.

4. **Formatting of tables and figures**
    - Ensure tables are formatted consistently (some captions are embedded mid-text).
    - Figures (e.g., Figure 1 and Figure 2) should be labeled and cited consistently in the text.
5. **Ethics and data availability**
    - In the main manuscript, the *Data availability* statement says "Data will be made available on request," while in the submission form it says "All relevant data are within the manuscript and its Supporting Information." Please ensure consistency with PLOS ONE's data-sharing policy.
6. **References**
    - Check reference formatting for consistency (e.g., spacing, capitalization, and journal abbreviations).
    - Ensure all URLs (e.g., IntechOpen, FAO, census report) are valid and accessible.
7. **Language polishing**
    - A few minor grammatical issues:
        - "mastitis key risk factors" → "key mastitis risk factors"
        - "poor farms biosecurity" → "poor farm biosecurity"
        - "milk discards during treatment, culling and occasionally deaths" → "milk discards during treatment, culling, and occasional deaths"

Recommendation

Minor Revision – The manuscript is strong overall. With improvements in clarity, consistency, and formatting, it will be ready for publication.
