# Supplementary material for: Bovine mastitis epidemiology: Prevalence, risk factors, control program gaps and biosecurity recommendations to improve animal health in the Rwandan smallholder dairy farms
Source: PLoS One. 2026 Mar 6;21(3):e0329250. doi: 10.1371/journal.pone.0329250 (PMC12965528; doi:10.1371/journal.pone.0329250)
Supplement: Attachment — Submitted filename: Rebuttal letter addressing all comments.docx [file pone.0329250.s006.docx]

# Response to Reviewers

Manuscript title: **Bovine mastitis epidemiology: prevalence, risk factors, control program gaps and biosecurity recommendations to improve animal health in the Rwandan smallholder dairy farms.**

Journal: PLOS ONE

Dear Academic Editor and Reviewers,

## We would like to thank you very much for the time and effort you devoted to reviewing our manuscript and for providing constructive feedback that has helped us improve the clarity and quality of our work. We have carefully considered all the comments and addressed each point in the revised version of the manuscript.

## Below, we provide a detailed response to each comment raised by the editor and reviewers. All changes have been incorporated into the revised manuscript and are highlighted using track changes in the file labeled 'Revised Manuscript with Track Changes.' Line numbers refer to those in the revised version.

## Editorial Comments

**Comment 1: *Please ensure that your manuscript meets PLOS ONE's style requirements, including those for file naming.***

**Response:** We reviewed the journal’s style guidelines and adjusted the manuscript accordingly. Please refer to the final revised “**Manuscript**”.

**Comment 2: *In the ethics statement in the Methods, you have specified that verbal consent was obtained. Please provide additional details regarding how this consent was documented and witnessed, and state whether this was approved by the IRB.***

**Response:** Formal IRB review was not required because the study did not involve the collection of human or animal biological samples or any sensitive personal data. Instead, ethical approval was obtained from the relevant administrative authorities at the district level in Nyabihu and Musanze, Rwanda, in accordance with Rwanda’s national research ethics guidelines. The study’s objective and procedures were explained to all participating farmers. As many participants were unable to read or write, verbal informed consent was obtained prior to each animal clinical assessment and interview, and the process was witnessed and documented by a local veterinary or extension officer. The revised *Materials and Methods – Ethical Considerations* section now includes this clarification and confirms compliance with the Declaration of Helsinki principles. (Lines 107–115).

**Comment 3: *We note that your Data Availability Statement is currently as follows: 'All relevant data are within the manuscript and its Supporting Information files.'***

**Response:** Revised for conciseness and consistency with PLOS ONE’s requirements. Additional Excel data and the questionnaire used for data collection have been uploaded as a zip file in supporting information (Line 410).

**Comment 4: *Data sharing policy: All research data must be made freely accessible upon acceptance unless ethical restrictions apply.***

**Response:** The full dataset has been submitted as supporting information.

**Comment 5: *Ethics statement: The ethics statement must appear only in the Methods section of the manuscript.***

**Response:** The ethics statement now appears only in the Methods section (Lines 107–115).

**Comment 6: *Copyright compliance: Figures using copyrighted map or satellite images (e.g., Google Maps) cannot be published unless permission is granted.***

**Response:** Permission was requested from ESRI, as we used ArcGIS to generate the map; however, we did not receive authorization for the copyrighted material used in Figure 1. Therefore, Figure 1 was removed from the manuscript, and the relevant geographical information was retained in the text.

**Comment 7: *Reviewer-suggested citations: You should evaluate any papers suggested by reviewers and cite them only if relevant.***

**Response**: All suggested references were evaluated, and only relevant ones were addressed.

**Comment 8: *Reference list accuracy: Review and correct your reference list, remove or justify any retracted papers, and note all reference changes.***

**Response**: The reference list was reviewed and corrected. No retracted papers are cited; capitalization and URLs were standardized.

## Reviewer Comments

**Comment 1: *Some sentences are long and could be made more concise (particularly in the Introduction and Discussion).***

**Response:** Long sentences in the entire manuscript were shortened for better readability (e.g., Abstract Lines 41–43; Introduction Lines 55–57, Discussion: 329-335).

**Comment 2: *Use uniform terms for cow hygiene (e.g., 'very dirty,' 'dirty’) currently mixed with 'muddy filled.'***

**Response:** A uniform hygiene scoring system ('Not dirty,' 'Slightly dirty,' 'Fairly dirty,' 'Very dirty') was applied (Lines 174–177) and removes confusion. The term 'mastitis control variants' was revised to “mastitis control supplies” (e.g., disinfectants such as iodine or chlorine dioxide)' (Lines 37–38).

**Comment 3: *Some odds ratios have extremely wide confidence intervals (e.g., OR = 11.02; 95% CI: 1.449–490.310). Mention this limitation briefly in the Discussion.***

**Response:** Wide confidence intervals were acknowledged as a limitation in the Discussion (Lines 367–369).

**Comment 4: *Ensure tables are formatted consistently (some captions are embedded mid-text).***

**Response:** All tables were reformatted for consistency, and captions properly aligned. Figure 1 was removed; Figure 2 was relabeled and cited correctly.

**Comment 5: *In the main manuscript, the Data Availability statement says 'Data will be made available on request.' Please ensure consistency with PLOS ONE’s data-sharing policy.***

**Response:** The Data Availability Statement was unified to read: 'All relevant data are within the manuscript and its Supporting Information.' The dataset has been attached.

**Comment 6: *Check reference formatting for consistency (e.g., spacing, capitalization, and journal abbreviations).***

**Response:** Reference formatting, capitalization, spacing, and URLs were checked and corrected.

**Comment 7: *A few minor grammatical issues (e.g., 'mastitis key risk factors' → 'key mastitis risk factors').***

**Response:** Minor grammatical issues were corrected (e.g., 'key mastitis risk factors,' 'poor farm biosecurity,' and 'occasional deaths') (Lines 21, 38, 63).

We believe these revisions have addressed all comments and significantly improved the manuscript. We sincerely thank the editor and reviewers for their valuable input and consideration.

Sincerely,

Dr. Blaise Iraguha (on behalf of all co-authors)

Rwanda Dairy Development Project, RDDP

[Irablais2000@gmail.com](mailto:Irablais2000@gmail.com)

**November 10, 2025**
